# Supplementary material for: Separate, separated, and together: the transcriptional program of the Clostridium acetobutylicum-Clostridium ljungdahlii syntrophy leading to interspecies cell fusion
Source: mSystems. 2025 Apr 29;10(5):e00030-25. doi: 10.1128/msystems.00030-25 (PMC12090709; doi:10.1128/msystems.00030-25)
Supplement: Document S1 — Analysis of data presented in Tables S14 and S15 and Fig. S2. [file msystems.00030-25-s0001.docx]

Supplementary Text for

**Separate, Separated and Together: the Transcriptional Program of the *Clostridium* *acetobutylicum-* *Clostridium* *ljungdahlii* syntrophy leading to interspecies cell fusion**

Noah B. Willis & Eleftherios T. Papoutsakis

**Supplementary Text Document 1 for the analysis of the data presented in Tables S14-S15 and Figure S2**

**Timepoint Overlap and KEGG Pathway Analysis of Differential Gene Expression in Type I and II Experiments**

All differentially expressed genes were classified by pathway according to the KEGG database and analyzed via simple functional enrichment using the hypergeometric test (53) to determine which KEGG pathways were statistically overrepresented in the differentially expressed gene list for each species, timepoint, and subsection (Table S14, S15). All genes differentially expressed in *C. acetobutylicum* and *C. ljungdahlii* at a given timepoint in one or both RNAseq comparisons, as well as the subfractions of genes that were differentially expressed at multiple timepoints are illustrated by the Venn diagrams of Figs. S2A and S2B. The major conclusion from the KEGG pathway analysis was that coculture significantly impacts gene expression of amino acid metabolism genes in both organisms in both the Type I and II experiments, especially biosynthesis of arginine, histidine, and tryptophan. Many other KEGG pathways were differentially regulated in each individual organism at different timepoints, but the impact on amino acid metabolism was seen in both organisms across all timepoints in both the Type I and II experiments.
