# Supplementary figures and images for: Separate, separated, and together: the transcriptional program of the Clostridium acetobutylicum-Clostridium ljungdahlii syntrophy leading to interspecies cell fusion
Source: mSystems. 2025 Apr 29;10(5):e00030-25. doi: 10.1128/msystems.00030-25 (PMC12090709; doi:10.1128/msystems.00030-25)

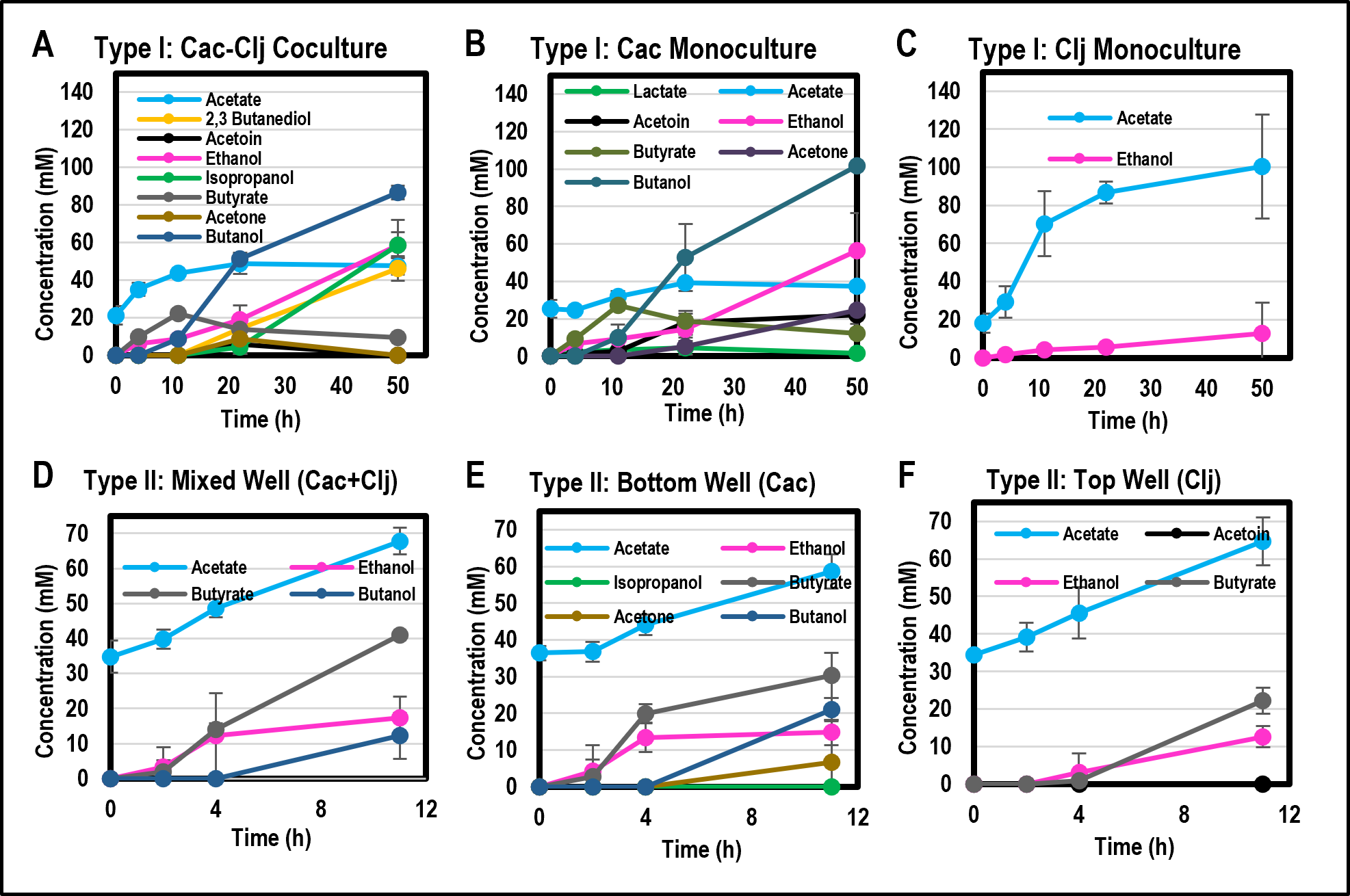

Supplement: Fig. S1 — Metabolite kinetics from type I and II RNAseq experiments. [file msystems.00030-25-s0002.tif]

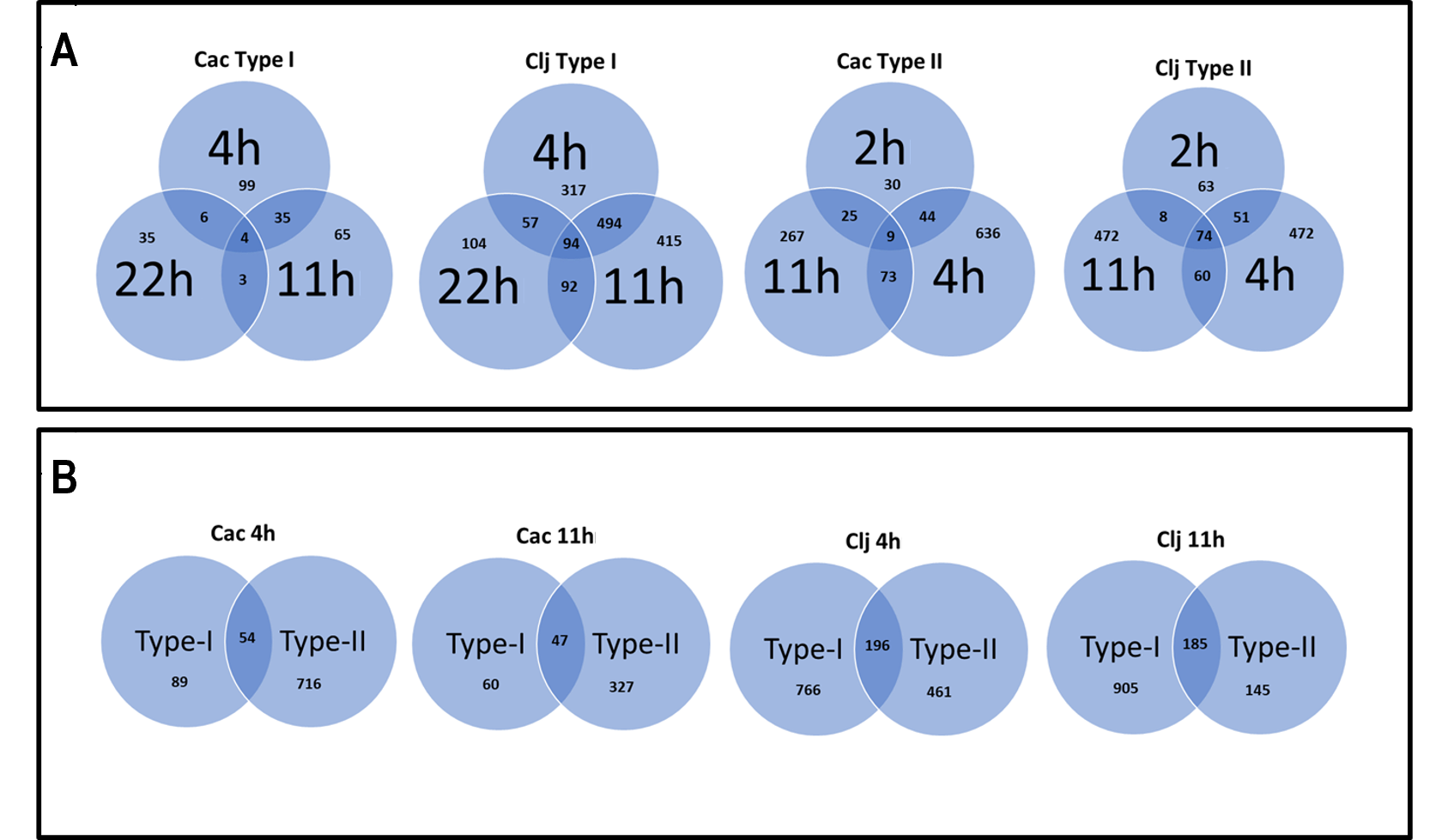

Supplement: Fig. S2 — Gene expression overlap across timepoints of type I and II RNAseq experiments. [file msystems.00030-25-s0003.tif]

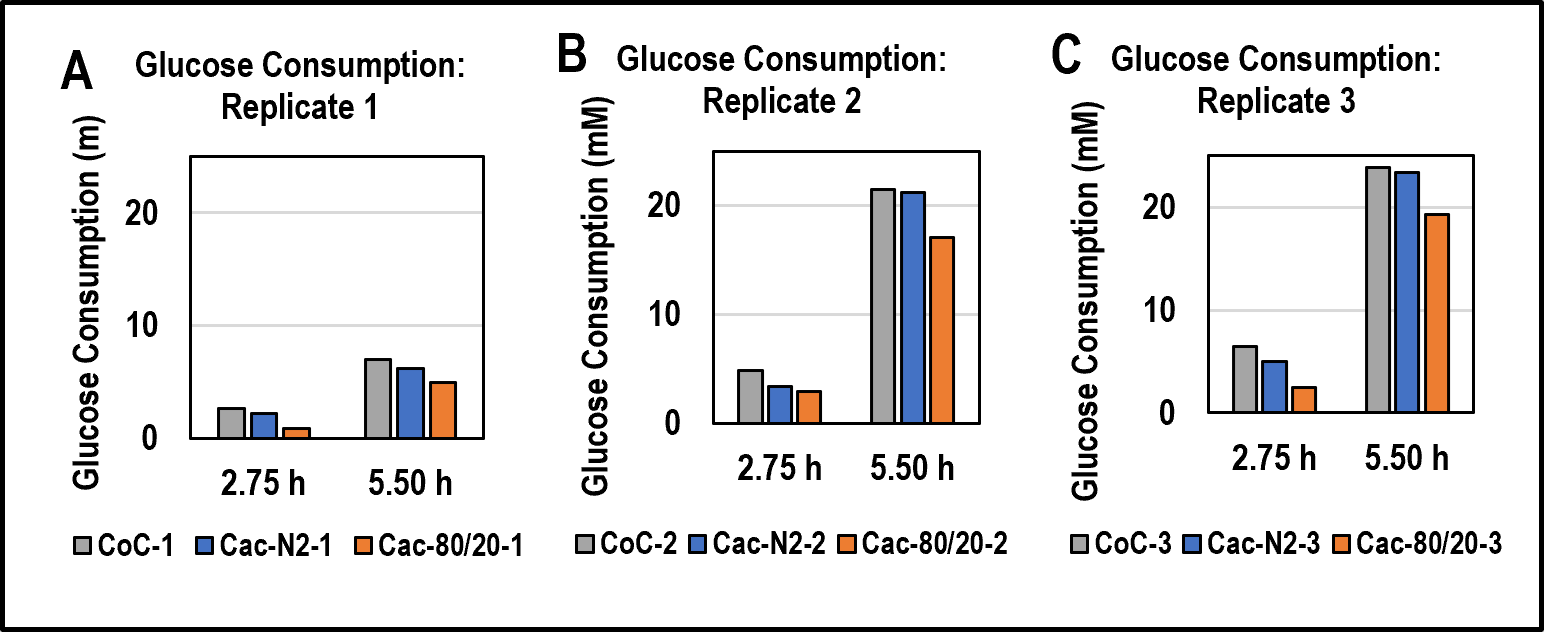

Supplement: Fig. S3 — Cumulative glucose consumption by C. acetobutylicum in response to different hydrogen environments. [file msystems.00030-25-s0004.tif]
