# Supplementary material for: Separate, separated, and together: the transcriptional program of the Clostridium acetobutylicum-Clostridium ljungdahlii syntrophy leading to interspecies cell fusion
Source: mSystems. 2025 Apr 29;10(5):e00030-25. doi: 10.1128/msystems.00030-25 (PMC12090709; doi:10.1128/msystems.00030-25)
Supplement: Legends — Supplemental figure legends [file msystems.00030-25-s0005.docx]

Figure S1: Temporal metabolite profiles. A) Type I C. acetobutylicum (Cac)- C. ljungdahlii (Clj) cocultures. B) Type I C. acetobutylicum monoculture. C) Type I C. ljungdahlii (monoculture. D) Type I unseparated C. acetobutylicum- C. ljungdahlii coculture . E) Type II bottom well (C. acetobutylicum) culture. F) Type II top well (C. ljungdahlii) culture.

Figure S2: A) Venn diagrams of differentially expressed genes at one or more timepoints for C. acetobutylicum (Cac) and C. ljungdahlii (Clj) in Type I and Type II experiments. B) Venn diagrams of differentially expressed genes in either the Type or Type II or both experiments at each timepoint in both C. acetobutylicum and C. ljungdahlii. Venn diagrams represent global picture of gene expression as described in Supplementary Document 1.

Figure S3: Cumulative glucose consumption at each timepoint for C. acetobutylicum (Cac)- C. ljungdahlii (Clj) coculture (CoC), C. acetobutylicum monoculture with N_2_ headspace (Cac-N2), and C. acetobutylicum monoculture with 80% H_2_ / 20% CO_2_ headspace (Cac-80/20). Three biological replicates (A, B, C) shown individually.
